# The Big Bang of Originality and Effectiveness: A Dynamic Creativity Framework and Its Application to Scientific Missions

**DOI:** 10.3389/fpsyg.2020.575067

**Published:** 2020-09-18

**Authors:** Giovanni Emanuele Corazza, Todd Lubart

**Affiliations:** ^1^Department of Electrical, Electronic, and Information Engineering, Marconi Institute for Creativity, University of Bologna, Bologna, Italy; ^2^Laboratoire de Psychologie et d’Ergonomie Appliquée, Université de Paris and Université Gustave Eiffel, Paris, France

**Keywords:** creativity, dynamic universal creative process, cosmology, scientific missions for space exploration, tightness and looseness, definition of creativity

## Abstract

This article introduces a theoretical framework to conceptualize the dynamics of the phenomenon of creativity, which is then applied to the specific case of scientific missions for the exploration of the universe. Static definitions of creativity are insufficient for this purpose, as they fail to describe states of creative inconclusiveness as well as the time and culture-dependent estimation of the value of the outcomes of a creative process; therefore, a dynamic definition of creativity is introduced, justified, and adopted to build a dynamic creativity framework. Within this framework, creativity episodes are shown to be mutually interconnected through several mechanisms (past and future concatenation, estimation, and exaptation), to form a dynamic universal creativity process (DUCP), the beginning of which can be traced back to the Big Bang of our universe. The DUCP entails several layers of complexity (material, biological, sociocultural, and artificial), showing that creativity is not only a psychological construct for humans but rather a unifying cosmological principle. Context embeddedness is discussed in-depth, introducing a taxonomy based on the concepts of tightness and looseness as applied to conceptual space and time. This theoretical framework is, then, applied to the discussion of the design, realization, and operations of scientific missions for the exploration of the universe, taking as a reference the terminology adopted by the European Space Agency.

## Introduction: The Dynamic Creativity Framework

We live in a world of uncertainty, dynamically evolving at a very fast pace (Rosa, 2003; Corazza et al., 2010; Feather, 2013), and creativity is arguably the engine of this fundamental unpredictability. It should, therefore, appear to be paradoxical that most definitions of creativity appear to be static, involving a definitive assessment of originality and effectiveness or similar statements, such as novelty and utility (Stein, 1953; Mayer, 1999; Parkhurst, 1999; Runco and Jaeger, 2012; Simonton, 2012; Martin and Wilson, 2017). We believe that basing a theoretical framework that should embrace the entire phenomenon of creativity on a static definition is clearly insufficient; it would be like trying to understand the plot of a thriller movie only through the final scene where the culprit is judged and jailed. A sense of extreme dissatisfaction would surge, and questions would be immediately raised: “What happened before the trial? What led to this end result? Could we please know the entire story?” In other words, in order to understand a dynamic, intriguing, and complex phenomenon, we need to be concerned with the entire *process* underlying the phenomenon itself, and not with a snapshot judgment of part or all of its outcomes. One might argue that the same standard definition of creativity as requiring originality and effectiveness (Runco and Jaeger, 2012) applies also to the creative process; however, this is unfortunately not the case for two very important reasons. First, in the course of any creative process which constitutes a real challenge, there are many instances of failure, in which the solution one is seeking is not found: under the static standard definition, these phases (which can also take very long time) could not be defined as “creative,” because neither originality nor effectiveness could be identified, and correctly so! This is a crucial problem, because in fact these temporary failures, which we identify as states of *creative inconclusiveness* (Corazza, 2016), are fundamental steps that all creative processes and persons must go through and show that they can be overcome. They are the very essence of the creative exploratory path, as shown very clearly by the example of one of the most prolific inventors of all times, Thomas Alva Edison (Edison, 1948; Wills, 2007). As a matter of fact, it has been shown that the history of artistic and scientific genius was paved by persistence and resilience in difficult times (Galton, 1869; Albert, 1983; Simonton, 1984; Eysenck, 1995). Evidently, this applies also to human creativity involved in scientific missions for the exploration of the universe. The second reason why a static definition of creativity is not able to capture the reality of a creative process is that no one, irrespective of his/her level of expertise on a knowledge domain, is entitled to give a final judgment on the originality and effectiveness of a product pertaining to that domain. Any assessment will always be subjective and partial: even the seemingly most objective measures such as uniqueness of a response (Wallach and Kogan, 1965) are in reality dependent on the sample of subjects participating to the experiment or analysis. Vice versa, in reality, any judgment depends on the entire context, an umbrella under which we classify the point of view of the judge and the surrounding culture, social space, and time epoch. Indeed, there are many cases of great creators who were not appreciated during their time: one example that stands out is certainly that of Vincent Van Gogh, who did not think much of himself as a painter and who sold only a single one of his works in the course of his lifetime (Van Gogh, 1978). Perennial glory for him would only come posthumously. To realize this fundamental fact allows us to grasp the true meaning of the *pragmatist maxim* by Peirce (1992–1999, p. 132): “*Consider what effects, which might conceivably have practical bearings, we conceive the object of our conception to have. Then, our conception of those effects is the whole of our conception of the object*.” In other words, the estimation of a creative idea entails the conception of all of its effects that might have practical bearing on reality: a dynamic, future-oriented, and never-ending exercise.

For these two reasons, given the necessity to account properly for creative inconclusiveness and the subjectivity of any judgment on the outcomes of a creative process, neither of which is captured by a static definition, a complete theoretical framework aimed at describing the entire phenomenon of creativity must be based on a dynamic approach (Beer, 2000; Beghetto and Corazza, 2019). This entails a dynamic definition of creativity, one that is able to subsume both instances of creative achievement and creative inconclusiveness and that should allow all the sociocultural variability that is intrinsic in the phenomenon (Glaveanu et al., 2019). To the aim of bridging these gaps, we adopt here a dynamic definition of creativity which is an evolution of the one presented in the work of Corazza (2016), according to which creativity requires *potential* originality and effectiveness. It should be clear by comparing this definition with the static one that the only difference lies in the insertion of the qualifier “potential” inside the definition, which applies to both originality and effectiveness. It is this single word that transforms a static picture taken by a photographer-judge into a dynamic process in which uncertainty dominates, but high levels of creativity can be attained by producing the conditions of high potential for possible future achievements of the goals of the process, or even serendipitous findings. The evolution of the definition presented in the work of Corazza (2016) that we propose here renders explicit the fundamental role of context in determining both the process itself and its interconnection with all of reality: *creativity is a context-embedded phenomenon requiring potential originality and effectiveness*. Context should be intended in its most general sense; in this article, it can be as vast as the universe but also as specific as a microscopic situation experienced by a specific being in a determined time instant. It can represent the different phases in the design and operation of a scientific mission for the exploration of the universe, as we will discuss later. Context embeddedness represents the fact that the resources, the affordances, the goals, the assessment criteria, and the sociocultural implications of a creative process all depend and cannot be isolated from the context in which they are displaced. Indeed, isolating a creative process from its context would be similar to studying the orbit of the Earth in the absence of the Sun: the solutions would be far from reality. In most cases, it will be the context in which the process is embedded that generates the presuppositions according to which the same outcome of the process can be considered either inconclusive or a creative achievement. We identify the *dynamic creativity framework* as the theoretical explanatory construction that descends from the adoption of the above dynamic definition of creativity.

## Big Bang and the Dynamic Universal Creativity Process

### Creativity Episodes

**Figure 1:**
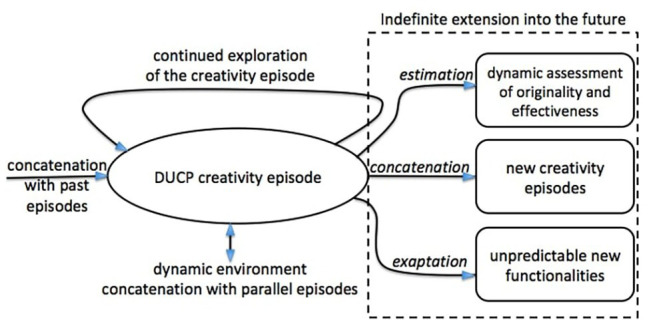
A dynamic universal creativity process (DUCP) creativity episode and its mechanisms for extension into the future.

Along the lines of Corazza (2019a), we define a specific instance of a creative process as a creativity *episode*. Under the dynamic framework, creativity episodes can be studied singularly for reasons of practicality but in reality have no rigidly defined ending and have indefinite connection to the past, as their influence extends indefinitely in time. All creativity episodes are interconnected, and as seen from a macroscopic point of view, they form a single overarching process which we identify as the dynamic universal creativity process (DUCP; Corazza, 2019a). Let us see the details of this fundamental observation pertaining to creativity episodes, with the help of Figure 1, which is an evolution of Fig. 17.1 from Corazza (2019a).

First of all, creativity cannot exist *ex nihilo*: the only extant possibility is to gather material, information, and knowledge from the past and use it in a way never attempted before, knowing that what will come out will emerge out of this previous legacy and may not be reducible to it. This means that a current creativity episode is concatenated to those previous episodes that produced the outcomes that have now become our ingredients. And, the chain into the past will continue indefinitely until a sort of DUCP origin is found. We shall return shortly on this ontological point.

Second, once the creativity episode is activated, there is no predetermined time limit to its duration, even though there may be many practical reasons why it may be desirable to set a maximum duration. But this limit is not intrinsic: the search for alternative ideas can continue indefinitely, both if we are in a state of creative inconclusiveness (no results deemed worthy have yet been found) and if we or the society around us can claim a creative achievement! This may sound surprising, but it is actually the trademark of great creators: never be satisfied by the first idea that appears fit. Keep refining or challenging your results: avoid early closure and develop a high tolerance of ambiguity (Zenasni et al., 2008). In essence, a creativity episode on a worthy focus can potentially continue indefinitely in time and deliver several outcomes along the way.

Third, while an actor or a team of actors is engaged in a creativity episode, other actors in the universe may be confronting similar endeavors: the interactions between these teams, either in the form of collaboration or of competition, form an indefinite extension in the action space of a specific creativity episode.

Fourth, suppose now that the subject creativity episode produces outcomes that are offered to the outside world, there are at least three mechanisms according to which the impact of these episodes can extend indefinitely into the future, as illustrated in Figure 1. The first mechanism is *estimation*: the dynamic extraction of the potential originality and potential effectiveness of a creative product entails that one imagines all possible values, in all possible futures, under all possible perspectives the product might have, as expressed by the pragmatist maxim mentioned above. Clearly, it is not a task that can be considered to be finished with a fixed amount of time and energy. This implies also that no one should ever be so arrogant to claim that his/her assessment of the creativity of the product is the final judgment, no matter the level of expertise of the judge. The second mechanism is *concatenation* into the future: just as the creativity episode under consideration took information from the past as an ingredient, the current outcomes may become ingredients of further episodes taking place in the future. And if, by chance or virtue, in the course of any of these future episodes, it is found that our episode under study produced outcomes that turn out *a posteriori* to have seminal value, our estimation of the current episode will have to be dynamically refined. In other words, the assessment of originality and effectiveness of past episodes is also dynamic. Finally, the third mechanism thanks to which the dynamic evolution in time of the impact of the outcomes of a creativity episode is indefinite is *exaptation* (Gould and Vrba, 1982): the possibility that an outcome of a creativity episode acquires in the future a totally new functionality that was not planned nor realized at the time of its generation. There are many instances of exaptation in the history of the arts, science, and technology, and so many more in biology, which is the domain in which the term was actually coined; not to be confused with adaptation. Whereas Darwinian adaptation foresees the evolution of an organism *via* DNA modifications and *a posteriori* discovery of higher aptness to the environment (Darwin, 1859), exaptation entails the search and discovery of new functionalities *with the same DNA*. The arguably unlimited power of exaptation becomes evident after reflection on technological evolution (Andriani and Cattani, 2016; Garud et al., 2016).

Clearly, the three mechanisms for extending the reach of a creativity episode into the future, namely estimation, concatenation, and exaptation, are non-orthogonal conceptual categories, so it is useful to clarify their main differences to justify their separation in the theoretical framework. Estimation refers to the appreciation of the properties of the outcomes of a creativity episode and does not necessarily lead to a new episode. Concatenation takes a past achievement as it is, with no concern for its estimation in all of its possible meanings, and exploits it as an ingredient for a new creativity episode. Finally, exaptation is the result of an effort to switch the functionality and meaning of an outcome, producing an explicit drift away from the objectives that drove the original creativity episode. As an example, let us consider the invention of the smartphone, a creativity episode that has had dramatic impact on the course of development of *Homo sapiens* in a relativity short amount of time (first models appeared around 2005). The estimation of the originality and effectiveness of the smartphone is continuously evolving with the number of possible applications that can be installed: present estimates indicate that there exist about 2 million software programs in each of the major application stores for smartphones. In terms of concatenation, the immediate step has been to take the principles and functionalities of the smartphone and transfer them to wearable devices, such as the wrist watch or the glasses. So far, the success of these devices is still limited, but they are likely to become customary accessories in the future. Finally, in terms of exaptation, it is to be noted that the main functionality of the smartphone in its initial conception was still that of a telephone augmented by side functionalities. But integrating one (or more) high resolution digital camera in the device unexpectedly turned the mainstream usage of the device from voice to images, so much so that the largest producers of cameras in the world soon became the smartphone manufacturing companies.

### Interconnecting Creativity Episodes Into a Universal Process

Given the above discussion, it should appear clearly that creativity episodes can be studied in isolation for practical purposes, but they are really part of an indefinite flow which interconnects them all, and this is the essence of the DUCP concept. The various models for creative processes that have been proposed (e.g., Wallas, 1926; Mumford et al., 1991; Lubart, 2001; Kaufman and Baer, 2004; Sternberg, 2006; Corazza and Agnoli, 2015) fulfilled the goal of describing with variable levels of detail the development of *a single creativity episode*. However, due to the intrinsic dynamicity of the phenomenon, these episodes are never effectively concluded nor disjoint, as discussed above. Indeed, the extant and undeniable interconnectivity between creativity episodes may in fact be one of the *strongest reasons for advocating a dynamic approach in creativity studies*. This brings us to the definition of the DUCP, as follows: “*The active ensemble of all creativity episodes in the course of cosmic evolution*.” The ensemble of all creativity episodes should be visualized as a tree-shaped structure of interconnected creativity episodes, each with its multifold creative potential that grows exponentially throughout history (Lehman, 1947; Enquist et al., 2008). The ensemble is active not only because it is continuously growing but also because concatenation with past creativity episodes changes the creative potential of those ancestors, perhaps changing what was considered to be a mediocre achievement into a seminal milestone!

### The Origins of Creativity in Our Cosmos

Now the ontological question is: when did the DUCP process begin to exist? If we remain within the realm of human action, it appears immediately that we cannot limit our search within *Homo sapiens*, because evident creativity episodes were enacted by hominid ancestors: remains of a stone-tool industry have been found in Kenya at the Lomekwi 3 site in West Turkana, Kenya, as far back as 3.3 million years ago (Harmand et al., 2015). Given the prehistoric evolutionary stage of these hominids, it is natural to ask whether humans are the only beings that can be accredited with creative behavior: the answer is absolutely not. Reflecting on the phenomenon of the emergence of life on Earth (Judson, 2017), it appears practically impossible to surpass this astonishing novelty in terms of its potential for originality and effectiveness. Once this fact is realized, one becomes open to find creativity episodes in animals (Kaufman and Kaufman, 2004), in plants (Gianoli and Carrasco-Urra, 2014), in monocellular organisms (Nakagaki et al., 2000), arguably in artificially intelligent agents (Colton et al., 2009; Brynjolfsson and McAfee, 2014), and even in inanimate matter. The latter point is surprising and must be discussed: how is it possible that inanimate matter be considered “creative”? How could matter produce outcomes that are characterized by potential originality and effectiveness? The answer to this question enjoys a theoretical framework of its own, which is that of complexity and complex systems. The work by Ilya Prigogine, the 1974 Nobel prize for chemistry, focused on the dynamics of dissipative systems which are far from equilibrium, was the key to the start of this extremely important line of thought (Prigogine, 1967, 1996). Indeed, what Prigogine has shown is that in these conditions, the evolution of a non-biological system can have trajectories that are fundamentally unpredictable. By “fundamentally,” it is intended that this unpredictability is not due to our inability to produce a mathematical model, but because of the intrinsic impossibility to predict the course of their dynamic evolution. It is this unpredictability that makes these trajectories a form of “creative achievement,” in the sense that they are original and effective, and this allows matter in the universe to be transformed in a positive way and not toward complete disorder (entropy), as the second theorem of thermodynamics would seem to imply. Indeed, our universe is a dissipative open system, the equilibrium of which was dramatically disturbed by the first impulse, the Big Bang, a gigantic surge of energy, the consequences of which are still evolving after some 13.7 billion years, in the course of which nearly 2 trillion galaxies were formed (Conselice et al., 2016), each with billions of stars, and each star potentially surrounded by planets. Note that creativity in nonlinear, dynamic, complex systems is an active research domain of its own (Ambrose, 2014; Loreto et al., 2016; Gabora, 2017). The initial conclusion to this discussion is the following, perhaps surprising, statement: *the origin of the DUCP is the Big Bang*, after which an indefinite concatenation of creativity episodes emerged at the material layer, at the biological layer, and at the sociocultural layer of complexity. This cosmological view of creativity is very much in line with the process philosophy developed by Alfred North Whitehead (Corazza, 2020) in “Process and Reality” (Whitehead, 1978/1929, p. 21), whereby creativity is elected to be the ultimate metaphysical principle, thanks to which the multitude of elements in the universe come together, moment by moment, to form instantaneous reality: “Creativity is the universal of universals, characterizing ultimate matter of fact. It is the ultimate principle by which the many, which are the universe disjunctively, become the one actual occasion, which is the universe conjunctively.”

### Creativity Beyond Humans?

The above theoretical framework foresees, therefore, that the creativity of humans represents but a small fraction of the DUCP, albeit the most significant one, as it is characterized by intentionality and intelligence: for this reason, we classify its creativity in the *strict-sense*. For an in-depth discussion about the rationale for allowing also a *wide-sense* view on creativity, one that does not necessarily require intentionality, extending the discussion to the biological layer, the material layer, and the artificial layer, the reader is referred to Corazza (2019a). For the purposes of this article, it suffices to say that this is in line with those approaches which have as a goal the *unification* of knowledge across different disciplines for the macroscopic understanding of cosmic evolution (Wilson, 1998; Henriques, 2003, 2011; Chaisson, 2009; Kauffman, 2016), as opposed to its segmentation into non-communicating silos. In other words, adopting this theoretical framework affords the possibility to create links between the development of originality and effectiveness across multiple levels of reality.

However, we should not leave this point without clarifying that attributing creativity to the material and biological layers does not violate the fact that creativity should be interpreted as a sociocultural category. It is still a human observer that understands and interprets the emergence of fundamentally unpredictable novelty in open physical or biological systems as creative phenomena. By the same token, emergence is a sociocultural category: the theory of complex systems is a symbolic framework of explanation developed by humans, the meaning of which can only be extracted through the use and within the boundaries of our extant culture.

We will focus the rest of this work on the sociocultural layer and on *Homo sapiens*, knowing, however, that we are immersed in a universe that is also evolving creatively toward growing levels of order.

## Creativity in Context: Tightness vs. Looseness

Considering the sociocultural layer of complexity in the DUCP, we intend here to discuss the characteristics of the context embedding the creative process and how they produce variable levels of potential originality and effectiveness. In other words, we are trying to answer the question: how can we classify the conditions that most significantly affect human creativity? As it turns out, the answer is multifold, and it depends on sociocultural variables that move along an axis going from maximum *tightness* to maximum *looseness* (Gelfand et al., 2011; Gelfand, 2012). Briefly, a tight society is one where there are very stringent norms, in which there is no tolerance for breaking the rules, in which behavior is encoded and monitored, not only by institutions but also by fellows. A paradigmatic example of a tight society might be the Republic of Singapore. At the other extreme, a loose society is one where norms are flexible and weakly applied, where there is tolerance for errors and violations, and in which behavior is quite free and socially liberated. A good example of such a society is, perhaps, given by New Zealand. It has been shown (Gelfand, 2012) that tightness and looseness are indeed new variables with respect to the more classic cultural dimensions such as collectivism and individualism and that they can be used to explain many societal characteristics, also in terms of innovation potential (Harrington and Gelfand, 2014). Here, we will use these concepts in a different way by applying them to two orthogonal dimensions of time and space.

Suppose that the creativity episode under consideration can be classified to belong to a certain conceptual or semantic space (Newell and Simon, 1972; Perkins, 1992; Boden, 2009), containing the knowledge that is relevant to the domain, including problems, constraints, and solutions, and expandable in view of present and future innovations (Kauffman, 2016). This conceptual space *S* can itself be tight or loose. A tight conceptual space is one where there is only the possibility for a single correct solution, at most with few variations on the theme, with many constraints, and where is very little or no tolerance for ambiguity and mistakes. In the terminology introduced by Perkins (1992), this would correspond to a Homing space, for which the structure of the problem indicates in itself the solution, allowing for an optimization of a response. On the contrary, a loose space is one where many alternatives are possible, with little or no preconditioning on the outcomes of the process, with ample possibility to accept paradigm shifts, and high tolerance for ambiguity. Referencing again to Perkins (1992), this would correspond to a so-called Klondike space, in which the most productive search is performed as an exploration of an unstructured space (see also Boden, 2009). It may be useful to fix our minds on two examples. Solving a mathematical problem in a new way is a creativity episode embedded into a tight space: there is only a single correct solution to the problem, and all the alternative procedures that one could devise must be conceived under the tight constraint of step-by-step correctness. On the other hand, consider writing a novel in a new genre: this is a goal that generates a creativity episode embedded in a loose context; the conceptual space is ill-defined (what do we mean by “new genre”?), and there are an indefinite number of possible outcomes, the value of which can only be seen *a posteriori*, because it is essentially unpredictable and highly dependent on who will be called on to judge it. In terms of the cognitive components of creativity, it should be evident that convergent thinking (Cropley, 2006) appears to be more fit in a tight space, whereas divergent thinking (Runco and Acar, 2012) would seem to belong to a loose space. However, appealing as these connections might seem, we should avoid building a one-to-one correspondence between the context characteristics and the ensuing cognitive components, for two reasons. First, irrespective of the context, any creative process will always use a combination of convergent and divergent thinking components, depending on whether one is defining the focus of attention, gathering relevant information, generating ideas, assessing outcomes, etc. Second, context produces a situation, an environment in which the actor operates according to his/her thinking style, but there is no cause-effect relationship between context and thinking components. This can lead to variable levels of accord or mismatch: for example, using divergent thinking in what society considers a tight space might lead to inefficiency, but perhaps also to the breaking of a consolidated paradigm. On the other hand, preferring convergent thinking in a loose context is clearly possible even though it might limit one’s freedom of thought and action, and perhaps be classified as boring behavior and/or personality.

Now let us consider the time variable *T*. As we have discussed before, under the dynamic creativity framework leading to the DUCP, there are no intrinsic constraints to the time duration of a creativity episode, so even this variable becomes part of the description of the context in which the process is embedded. Tight time *T* means that the context imposes stringent specifications on the time interval within which results are expected to come out of the process, and there is little or no tolerance for delay. Indeed, in extreme conditions, a delay could endanger one’s life, so much so that adhering to the time constraints becomes a matter of survival. Delay could also be severely punished by institutions. At the opposite end, loose time *T* means that there are ample periods of time during which outcomes can be produced, estimated, exapted, and concatenated. Planning is not at a prime, and there is ample tolerance for delays. The introduction of the concepts of tightness and looseness in the time dimension can be linked to the line of research related to the effects of time pressure on creativity (Amabile et al., 2002; Baer and Oldham, 2006). A sort of implicit theory exists about the fact that high time pressure, hence tightness in the *T* dimension, would lead to more creative solutions. A paradigmatic example would be that of the Apollo 13 mission in 1970, during which an explosion occurred, damaging the air filtration system and building carbon dioxide in the cabin. This was a clear life-endangering problem to be solved in extremely tight *T*. All NASA engineers, scientists, and technicians started to work on the problem, producing a solution based on the same material available onboard. The solution was inelegant and far from perfect, but it worked and saved three lives. However, as pointed out by Amabile et al. (2002), it would be incorrect to directly extend the validity of such examples to the more general context of the workplace. Indeed, it has been shown that having uninterrupted quiet time during specified periods every day can lead to higher creativity and wellness in the workplace. When time pressure cannot be avoided, it is, in any case, useful to make coworkers feel as if they are in a “mission,” so that they share a common fate (Amabile et al., 2002). It is interesting to note that the relationship between tightness of time *T* and the creative potential will not be linear but, in general, curvilinear: Baer and Oldham (2006) have found an inverted U shape, moderated by openness to experience and support for creativity.

### Space-Time Quadrants in the ST Plane

By crossing these two dimensions of space *S* and time *T*, each one varying from extreme tightness to extreme looseness, respectively, in the horizontal and vertical dimensions, four quadrants are formed in a conceptual ST-plane:

- Quadrant I: Tight S – Tight T (pure tightness)
- Quadrant II: Loose S – Tight T (hybrid looseness-tightness)
- Quadrant III: Loose S – Loose T (pure looseness)
- Quadrant IV: Tight S – Loose T (hybrid tightness-looseness)

As we will show in the following, these four quadrants correspond to very different contextual conditions, leading to quite different forms of potential for originality and effectiveness of creativity episodes. Let us discuss them following a trajectory that starts at Quadrant I, then on to II, IV, and finally III.

#### Quadrant I: Tight S – Tight T

In these conditions, context is tight both in space *S* and time *T*. The constraints are typically so strong that the actor is forced to search for the best solution to any problem he/she might face in the minimum possible amount of time. Pressure is high both in time *T* and in space *S*. There is no tolerance for ambiguity nor delay. This is actually the typical situation in which humans live, especially in the educational, academic, and professional environments. A test, such as for example an IQ test, produces the sort of constraints that can be properly mapped onto Quadrant I: correct answers are expected to be given within the allocated time. No tolerance exists whatsoever, and every mistake counts in lowering your score. Also pertaining to Quadrant I would be a situation such as the launch of a spaceship for human flight: planning is extremely precise down to the detail, no ambiguity is tolerated anywhere, and any mistake can lead to the loss of lives. It should be clear that in these tight space-tight time conditions, the potential for originality and effectiveness is in general quite low, given the high level of constraints and the strong punishments associated with failures. There is very little or no room for creative inconclusiveness. Those few individuals who, faced with an urgent and unforeseen problem, are able to solve it in a surprising way while remaining within the tight space-time boundaries of the context are usually considered to be geniuses.

#### Quadrant II: Loose S – Tight T

In these conditions, time remains tight but the constraints and expectations on the conceptual space are loosened or completely removed. We have a sense of urgency, time pressure is high, and there is little or no tolerance for delays; however, the problem we are facing is open-ended and allows a multitude of possible responses, the originality and effectiveness of which can only be judge *a posteriori*, because the scenario is unknown or at least ill-defined. The pressure on the actor is, perhaps, even larger, and the potential for originality and effectiveness is certainly higher than in Quadrant I. It is accepted that within the multiple responses that can be conceived in the conceptual space, not all of them will be successful, but there is sufficient freedom in order to search for remote solutions with high originality, albeit in a tight time frame. Clearly, the Apollo 13 incident that we mentioned earlier would fall in this Quadrant II category; let us give two additional examples that appear to fit well here. First, consider the classic Alternative Uses Test (Christensen et al., 1960) that is used in the majority of papers on creativity to measure the divergent thinking ability, one of the most important components of the creative thinking process, albeit not the only one. In these testing conditions, the task could be to produce all the possible alternative uses of a brick beyond the conventional in a few minutes, e.g., 3. The performance is measured in terms of fluency (number of responses), originality (typically scored by external judges), and perhaps flexibility (number of conceptual categories visited). This is an open-ended context, but the time dimension is extremely tight: as such, it belongs to Quadrant II. A second example refers to a space mission for exploration of the universe: suppose you are in a mission to Mars and you exited the base to explore the surrounding environment. You are equipped with multiple measurement tools, and your autonomy is limited to 15 min. You are the first human to ever explore this part of the red planet. This is an excellent example of a Quadrant II situation, one in which time is tightly constrained but space is extremely open, and indeed surprising discoveries are possible and even expected. However, some mistakes can be fatal, and certainly there is no tolerance in trespassing the allowed time limits: this will likely lead to a loss of life. This is the quadrant that, perhaps, best represents the context embedding the creative process in extreme conditions.

#### Quadrant IV: Tight S – Loose T

This Quadrant is somewhat dual to Quadrant II we just considered, also leading to a hybrid context in terms of tightness vs. looseness, but in this case, we loosen the time *T* constraints, whereas space *S* remains tight. Within this context, one is typically faced with a problem of high to very high complexity, perhaps unsolved by many years, decades, or even centuries. In this case, there is no expectation that a new solution will be conceived or discovered in a limited time frame, but if it were to be found, the value would be extremely significant. This quadrant can be considered to be the home of complex problem solving, which is notoriously considered to be an important part of intelligence. In terms of creativity pertaining to Quadrant IV, perhaps one of the most fitting examples is the activity of Henri Poincaré (Corazza and Lubart, 2019), so well described in his *Science and Method* book, which also gave input to the famous four-stage model of the creative process (Wallas, 1926). Another example can come in terms of creative planning to prevent future extreme conditions; even though they are not experienced in real-time, they must, however, be *anticipated* (Corazza, 2017a) in order for the design to be credible and successful.

#### Quadrant III: Loose S – Loose T

Finally, Quadrant III is the dual to Quadrant I, the loose context whereby both space *S* and time *T* are loosened. Embedding, a creative process in this context where constraints are, in general, very weak, allows the maximum freedom of exploration. Clearly, there are no guarantees that a creative achievement will occur, but there is ample tolerance for creative inconclusiveness. The potential for originality is at its highest level; the potential for effectiveness is variable and can also be quite low. Certainly, this quadrant can be considered to be the home of artistic creativity: *a priori*, there is no information about the form of the process outcomes, one can enjoy maximum freedom for probing alternatives, even in areas where there is no “problem” to be solved. The results are not expected to come within predetermined time limits, and recognition of their value could even occur posthumously. A paradigmatic example of creative activity in a context represented by Quadrant III is the painting career of Vincent Van Gogh, as previously recalled. Providing such a context in an educational environment will generate the best embedding conditions to nurture and develop one’s creative abilities and to strengthen one’s creative identity. Indeed, one mistake that society can make is to impose excessively tight time schedules to an activity that would be best to belong to Quadrant III, effectively moving it to Quadrant II.

As can be seen, the use of the concepts of tightness vs. looseness in association with conceptual space *S* and time *T* enables the introduction of a very clear taxonomy and possibility for classification of the context in which the creative process is embedded. The potential for originality and effectiveness is strongly influenced by the tightness vs. looseness of this context, and it is important to understand and classify these contextual conditions in order to ensure that the creative process is conducted in the most proficient way. Of course, the perception of the tightness and looseness of space and time is subject to individual and societal differences: for example, in a school environment, a math test to be carried out in a predetermined amount of time may be perceived as a very tight context by the average of the class but as significantly looser by a gifted student. This variation is actually the rationale for designing specific educational programs for the gifted.

Now our objective is to apply the concepts of the dynamic creativity framework, dynamic definition of creativity, DUCP, and ST-quadrants to an analysis of the creative process in the framework of designing and operating scientific missions for the exploration of the universe.

## Scientific Missions for the Exploration of the Universe: Contexts for Creativity

In this section, we intend to show how the design of a scientific mission for exploration of the universe follows a sequence of phases that can be mapped as a trajectory over the ST-quadrants discussed above. For each phase, an indicative estimate of the respective potential for originality and effectiveness is given. It is very important that these levels of potential are not confused with those required to establish a creative achievement; for example, if a creative process is embedded into a context with medium potential for originality and low potential for effectiveness, the probability to obtain creative achievements will be medium-low, but when this infrequent event occurs, this outcome will have to be characterized by high originality and high effectiveness.

**Figure 2:**
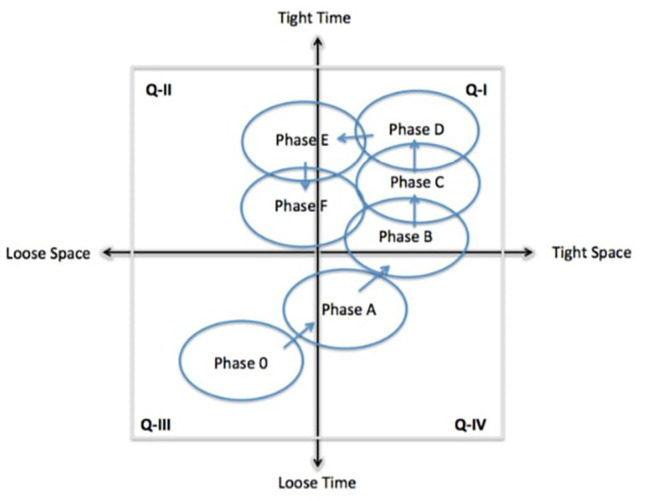
Mapping mission phases on ST-quadrants.

Following the classification adopted the European Space Agency (ESA, 2020), seven phases can be recognized in the identification, definition, and realization of a mission: *Phase 0*, Mission identification; *Phase A*, Feasibility; *Phase B*, Preliminary Definition; *Phase C*, Detailed Definition; *Phase D*, Qualification and Production; *Phase E*, Utilization; and finally *Phase F*, Disposal. Let us analyze these phases in terms of their mapping onto the ST-quadrants and the consequent implications on the potential for originality and effectiveness, with the help of Table 1 and Figure 2.

**Table 1:** Scientific mission phases, associated ST-quadrant, and creative potential.

Mission phase	Description	ST quadrant	Creative potential
Phase 0	Mission identification	III	Pot. originality: high Pot. effectiveness: low-to-medium
Phase A	Feasibility	III, IV	Pot. originality: medium Pot. effectiveness: medium
Phase B	Preliminary definition	I	Pot. originality: low-to-medium Pot. effectiveness: medium-to-high
Phase C	Detailed definition	I	Pot. originality: low-to-medium Pot. effectiveness: medium-to-high
Phase D	Qualification and production	I	Pot. originality: low Pot. effectiveness: high
Phase E	Utilization	I, II	Pot. originality: low-to-medium Pot. effectiveness: high
Phase F	Disposal	I, II	Pot. originality: low-to-medium Pot. effectiveness: high

In Phase 0, ESA opens a call for proposals for new science missions, addressed to the wider scientific community. This is quite an open-ended exercise which can be mapped onto Quadrant III. In fact, each call for missions can generate several dozens of responses from different academic groups, addressing areas as wide as fundamental physics, solar system structure, astronomy, etc. The general aim is to produce a broad spectrum of ideas and alternative concepts to be explored. The potential for originality in these proposals is quite high, and typically the potential for effectiveness can be classified as low-to-medium, because in-depth feasibility studies still need to be performed. In fact, the boldest mission proposals require typically the development of new technology, the feasibility of which cannot be guaranteed *a priori*. These proposals are assessed by ESA’s scientific advisory committees of experts, such as the Science Programme Committee, the Space Science Advisory Committee, the Astronomy Working Group, the Solar System Working Group, or the Fundamental Physics Working Group. Both originality of the mission and its preliminary effectiveness in terms of feasibility are taken into account in these evaluations. This pre-screening effort will produce a short-list of three or four candidates; for each retained proposal, a team formed by a scientist and an engineer is formed for a 1-year feasibility study. The time dimension *T* is still considerably loose: even though a deadline is established, the amount of available time is more than sufficient to work and explore without excessive time pressure. This study has, in particular, the objective to identify precisely any new technology that needs to be developed to make the mission possible, and therefore effective. The end results of all these Phase 0 studies are presented at ESA headquarters in Paris to ESA’s scientific advisory committees, which have to select and recommend those missions that should proceed to Phase A. It is typical that two or three missions are selected for a Phase A study. In conclusion, Phase 0 of a scientific mission for universe exploration can be largely classified as belonging to Quadrant III, with a high potential for originality and a low-to-medium potential for effectiveness.

Considering Phase A, aimed at establishing feasibility, the design of a mission can be awarded in the form of contracts to two competitive industrial teams. The purpose of having competition at this early stage is to allow for alternative solutions to come up and be contrasted with one another. This guarantees to keep up the level of originality while starting to focus down on effectiveness, both in terms of performance and cost. Each competing team must generate a preliminary design and a project plan specifying details about necessary spacecraft instruments, system and subsystem manufacturing, launch, orbital characteristics for spacecraft, time plan to reach target, and scientific operations to be carried out once the target is reached. All elements of the preliminary design must be accompanied by estimated costs. For any new technology identified, it is important to present at least a proof-of-concept. Overall, the potential for originality of Phase A, as compared to Phase 0, decreases to a medium level, even though proofs-of-concept can often lead to patents, while the potential for effectiveness grows to reach also a medium level. Phase A can be mapped onto a mixture of Quadrant III and Quadrant IV, given that the conceptual space *S* is narrowed down but still allows alternatives, whereas the time *T* is still loose given that no final decision has been made yet, and therefore, schedules are not as tight as they become in subsequent phases. Preliminary designs and project plans are compared and a decision is made on the specific mission design.

From the point of view of analyzing creative potential, Phases B (Preliminary Definition) and C (Detailed Definition) can possibly be discussed together: they are both concerned with narrowing down (in two concatenated stages) all engineering details in order to arrive at a complete design *on paper*. It should be noted that any error or mistake in the definition of a project will translate into significant losses of time and money when manufacturing and assembly will occur in Phase D, and therefore, they are to be avoided as much as possible. Indeed, a mistake here can easily translate into losing one’s job. This clearly tends to stifle creativity. On the other hand, design problems that might occur in Phases B and C may lead to creative problem solving, and these creative solutions can potentially lead to patents or invention disclosures. For these characteristics, potential originality can be considered to be low-to-medium, whereas potential effectiveness is medium-to-high, although no actual realization is attempted yet. In terms of mapping, Phases B and C progressively move the process into Quadrant I, where both conceptual space *S* and time *T* become tight.

Phase D concerns space qualification of all technologies, manufacturing of parts, assembly, and testing. In this phase, investments have all been decided and, therefore, schedules are precise and tight. Any problem has to be resolved as quickly and as correctly as possible. Virtually no room is allowed for innovations, but only problem solving upon necessity (typically not of a creative kind). Phase D is definitely a Quadrant I activity, a tight context with a very low potential for originality and a very high potential for effectiveness, leading to an overall low level of creativity.

Finally, we discuss Phases E (Utilization) and F (Disposal). When the actual scientific mission is carried out in Phase E, one would hope that everything will run as planned, but deviations due to small or large unexpected difficulties are bound to occur. Therefore, rapid remedies to these unforeseen events must be devised, often putting at risk the success of the entire mission. For this reason, Phase E is a mixture of Quadrants I and II: time *T* is tight, but depending on the situation, we might use known solutions, exploit risk mitigation plans, or if none of the above works we might have to devise creative alternatives. In all of these cases, the potential for effectiveness of the ideas involved is high, while the potential for originality is low-to-medium. Phase F, at the end of the mission lifetime, although it could appear to involve very little creativity, in reality reserves often quite a few surprises, given the fact that it is difficult to plan many years in advance and more often than not technologies can survive longer than planned. Also, there are many ways in which a mission can be brought to an end, depending on the level of space debris that is allowed (in general, it should be as low as possible). In essence, Phase F is also mapped onto Quadrants I and II, with low-to-medium potential for originality and high potential for effectiveness.

All these creativity episodes, with variable context embeddedness and levels of embedded creative potential (Corazza and Glăveanu, 2020), form a concatenation which can be interpreted as part of DUCP and describe a trajectory across the ST-quadrants. This discussion is summarized in Table 1, and the trajectory in Figure 2. It should be noted that this trajectory should be interpreted as a best practice, according to the process adopted by ESA. Nothing excludes the possibility for alternative trajectories to be established, but of course they would have to be justified with specific advantages. For example, forcing Phase 0 into Quadrant II, by imposing very stringent time schedules for the definition of innovative proposals is possible but it produces as a consequence that most of the proposals will be highly predictable, i.e., less original.

## Conclusion

Creativity studies are often focused on gathering and interpreting experimental data. This is a very important approach that should be accompanied and positioned with a comprehensive theoretical framework, one that affords macroscopic understanding and interdisciplinary associations. This article is aimed at providing such a theoretical framework: starting from the dynamic definition of creativity, introducing the concept of potential for originality and effectiveness, it is possible to describe a dynamic creativity framework, whereby creativity episodes enjoy indefinite time duration and are all interconnected into a DUCP. The universality of DUCP is literal in the sense that its beginning must be traced back to the Big Bang, and its development spans material, biological, and sociocultural layers of complexity. The creativity of humans is, therefore, not the only form of creativity that can be found in the universe, and scientific missions for the exploration of outer space should be interpreted as the honing of human creativity to appreciate, understand, and exploit the creativity of the universe. Considering the sociocultural layer of complexity, defining the concepts of tightness and looseness and applying them to the conceptual space *S* (the semantic and procedural domain for the search of ideas, actions, solutions, and decisions) and to T, the time-domain characteristics of the creativity episode, it is possible to identify four quadrants that describe significant contexts in which the creative process for humans can be embedded. The extension of the interpretation of these quadrants for the material and biological layers is possible, but it is left as future work. Exploiting this theoretical framework and this taxonomy, it is finally possible to analyze that various phases that are designated to manage and conduct a scientific mission for the exploration of the universe, and draw characteristic trajectories across the ST-quadrants. The importance of this work should be seen in terms of the fundamental role that creativity is going to play in the future stage of our societal evolution, identified as the Post-Information Society, characterized by drastic changes in the job market and business models. A future in which our capacity to be creative will be tightly connected with our wellbeing (Corazza, 2017b, 2019b).

## Author Contributions

GEC provided the theoretical framework. TL provided guidance and important references. All authors contributed to the article and approved the submitted version.

### Conflict of Interest

The authors declare that the research was conducted in the absence of any commercial or financial relationships that could be construed as a potential conflict of interest.
